# Postmarketing safety of bimekizumab for hidradenitis suppurativa: An early pharmacovigilance analysis using the United States Food and Drug Administration Adverse Event Reporting System

**DOI:** 10.1016/j.jdin.2026.01.007

**Published:** 2026-02-03

**Authors:** Simona A. Alomary, Nicole J. Baker, Temitayo Ogunleye, Susan C. Taylor

**Affiliations:** a Rutgers New Jersey Medical School, Newark, New Jersey; b Department of Dermatology, Perelman School of Medicine at the University of Pennsylvania, Philadelphia, Pennsylvania; c Sidney Kimmel Medical College at Thomas Jefferson University, Philadelphia, Pennsylvania

**Keywords:** bimekizumab, biologic agents, FAERS database, hidradenitis suppurativa, IL-17 inhibitors, pharmacovigilance

*To the Editor:* Hidradenitis suppurativa (HS) is a chronic, recurrent inflammatory disease of the skin, predominantly afflicting apocrine-gland rich areas of the body. It initially presents with painful nodules and abscesses that can form sinus tracts and fistulas in later stages.^1^ Treatment selection is related to disease severity, with refractory disease often warranting the use of a biologic agent. Biologic agents currently used include adalimumab (TNF-α inhibitor), infliximab (TNF-α inhibitor), secukinumab (IL-17A inhibitor), and more recently, bimekizumab (IL-17A and IL-17F). Bimekizumab was approved by the United States Food and Drug Administration for adults with moderate to severe HS on November 20, 2024. Although postmarketing safety studies exist for adalimumab, infliximab, and secukinumab, early real-world safety data have yet to be assessed for bimekizumab in the treatment of HS.^2^

The U.S. Food and Drug Administration Adverse Event Reporting System was queried for adverse events (AEs) related to bimekizumab using OpenVigil 2.1, an open-access pharmacovigilance interface.^3^ Reports were included if they listed “hidradenitis” as the reason for use, involved adults (≥18 years), and had an initial event date between November 20, 2024, and October 20th, 2025 (current date at time of analysis). 239 unique AEs were identified (Fig 1). Clinical characteristics of these AE reports are summarized in Supplementary Table I, available via Mendeley at https://data.mendeley.com/datasets/wxnmgt7khb/1.

**Fig 1:**
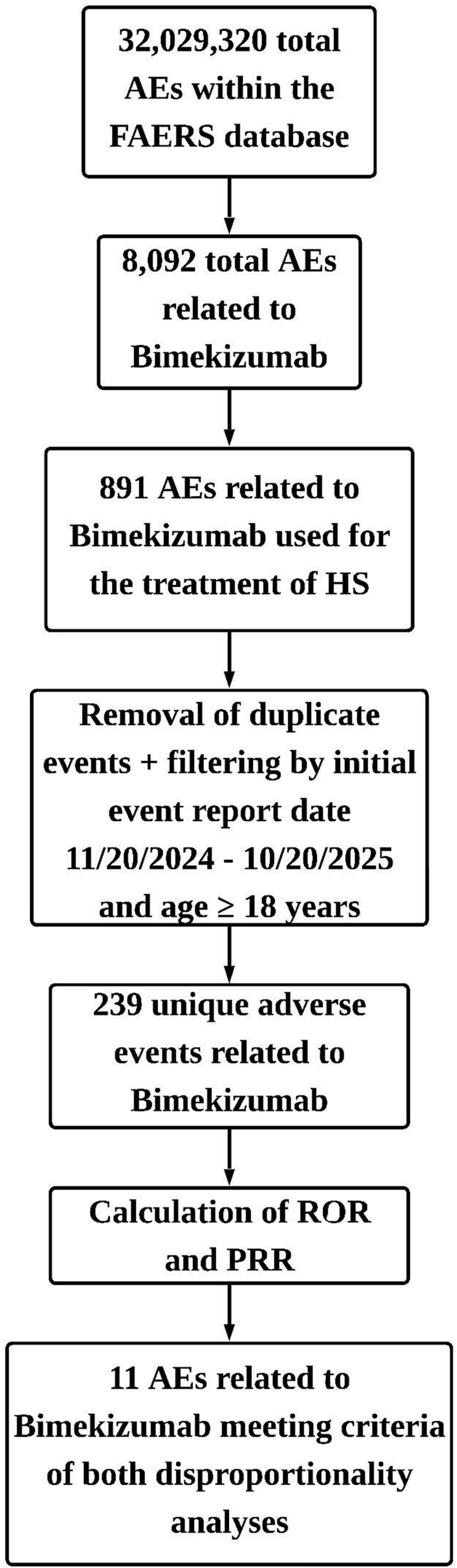
Workflow of adverse event (AE) extraction and analysis using the FDA Adverse Event Reporting System (FAERS), accessed through OpenVigil 2.1 Reports related to bimekizumab were filtered by indication (“hidradenitis,”) adult age, and report date. Duplicate events were removed, and disproportionality analyses were conducted. *AE*, Adverse event; *FAERS*, FDA Adverse Event Reporting System; *HS*, hidradenitis suppurativa; *PRR*, proportional reporting ratio; *ROR*, reporting odds ratio.

Disproportionality analysis using reporting odds ratios (RORs) with 95% confidence intervals and proportional reporting ratios with chi-squared statistics identified 11 significant AE signals (Table I). The strongest signals were observed for therapeutic response shortened (ROR 19.56), oral candidiasis (11.77), suicidal ideation (11.63), and nasal congestion (11.63). Additional signals included psoriatic arthropathy (9.77), hidradenitis (9.45), injection site pain (8.29), and depression (6.88). While AEs such as oral candidiasis and upper-respiratory tract symptoms such as nasal congestion align with established effects of IL-17-inhibition, signals such as therapeutic-response shortened likely reflect refractory disease.^4^ Although suicidal ideation had a higher ROR compared with other AEs, it was reported only 3 times (Table I). Given that Food and Drug Administration Adverse Event Reporting System does not include reliable exposure denominators, the total number of patients treated with bimekizumab is unknown, and ROR values should not be interpreted as event frequency. Additionally, psychiatric events such as depression and suicidal ideation may be related to underlying disease burden, as patients with severe HS have been found to have increased odds of depression and suicide when compared with controls.^5^

**Table I:** Significant adverse events (AEs) related to bimekizumab identified through disproportionality analyses, as previously described

Adverse event	Count (*N*)	ROR (95% CI)	PRR (χ2)
Hidradenitis∗	126	9.45 (6.79-13.14)	5.03 (214.78)
Injection site pain∗	29	8.29 (4.37-15.74)	7.41 (54.17)
Product dose omission issue∗	16	3.79 (1.89-7.63)	3.61 (14.29)
Inappropriate schedule of product administration∗	11	2.48 (1.18-5.52)	2.48 (4.94)
Incorrect dose administered∗	10	5.67 (2.14-15.06)	5.48 (13.03)
Depression∗	7	6.88 (1.99-23.70)	6.71 (9.98)
Therapeutic response shortened∗	5	19.56 (2.27-168.19)	19.17 (10.84)
Oral candidiasis∗	6	11.77 (2.36-58.69)	11.50 (11.34)
Psoriatic arthropathy∗	5	9.77 (1.88-50.66)	9.59 (8.16)
Suicidal ideation∗	3	11.63 (1.21-112.36)	11.50 (4.28)
Nasal congestion∗	3	11.63 (1.20-112.36)	11.50 (4.28)

All events shown met Evans criteria for probable association, which are defined below. Reporting odds ratios (RORs) with 95% confidence intervals (CIs) and proportional reporting ratios (PRRs) with chi-squared (χ^2^) statistics are displayed.

*AE*, Adverse event; *PRRs*, proportional reporting ratio; *RORs*, reporting odds ratio.

ROR = $\frac{\left(\frac{a}{c}\right)}{\left(\frac{b}{d}\right)}=\frac{ad}{bc}$.

$PRR=\frac{\frac{a}{a+b}}{\frac{c}{c+d}}$.

a = number of reports of adverse events for Bimekizumab.

b = number of reports of other adverse events for Bimekizumab.

c = number of reports of the same adverse event for all other drugs.

d = number of reports of all other adverse events for all other drugs.

∗ Signals meeting Evans criteria for probable association: PRR ≥ 2, χ2 ≥ 4, a ≥ 3, lower 95% CI > 1. $\chi^{2}=\frac{{\left(a\times d-b\times c\right)}^{2}\times \left(a+b+c+d\right)}{\left(a+b\right)\left(c+d\right)\left(a+c\right)\left(b+d\right)}$. 95% CI $=e^{\ln\left(ROR\right)\pm 1.96\times \sqrt{\frac{1}{a}+\frac{1}{b}+\frac{1}{c}+\frac{1}{d}}}$.

Given the range of potential side effects and the variability in individual responses to therapy, patients with HS on IL-17 inhibitors should be continuously monitored by a dermatologist who can provide guidance on dosing while monitoring for potential AEs. Inherent limitations of the Food and Drug Administration Adverse Event Reporting System database include voluntary reporting of AEs, lack of standardized reporting, and small sample sizes, which can lead to underreporting and potential biases within the data. This analysis is also limited to the initial 11 months following Food and Drug Administration approval of bimekizumab, reflecting an early postmarketing safety overview. As a result, delayed or long-term side effects may not be captured. Continued monitoring for bimekizumab is necessary in the future to characterize long-term AEs.

### Declaration of generative AI and AI-assisted technologies in the writing process

Not applicable.

## Conflicts of interest

Dr Taylor has served as a consultant, advisory board member, and/or speaker for AbbVie, Arcutis, Armis Scientific, Avita, Beiersdorf, Biorez, Bristol-Myers Squibb, Cara Therapeutics, Dior, Eli Lilly, EPI Health, Evolus, Galderma, GloGetter, Hugel America, Incyte, Johnson & Johnson, L’Oreal USA, MedScape, MJH LifeSciences, Pfizer, Piction Health, Sanofi, Scientis US, UCB, and Vichy Laboratories. Dr Ogunleye has served as a consultant, advisory board member, and/or speaker for Beiersdorf, MJH LifeSciences, Dermatology Times, and Health Central. Authors Alomary and Baker have no conflicts of interest to declare.
